# Complete Genome Sequence of Brevundimonas diminuta ATCC(B) 19146

**DOI:** 10.1128/MRA.00083-19

**Published:** 2019-03-28

**Authors:** Qian Liang, Wenwen Yu, Ya Shi, Hong Li, Xu Fang, Huan Chen

**Affiliations:** a Key Laboratory of Microbial Technology and Bioinformatics of Zhejiang Province, Zhejiang Institute of Microbiology, Hangzhou, Zhejiang, China; b China National Accreditation Institute for Conformity Assessment, Beijing, China; University of Maryland School of Medicine

## Abstract

Here, we describe the complete genome sequence of the Brevundimonas diminuta ATCC(B) 19146 strain, which contains 3,375 protein-coding genes and 61 RNAs within its 3,551,819-bp-long genome. The genome consists of a circular chromosome.

## ANNOUNCEMENT

Brevundimonas diminuta ATCC(B) 19146 is a small Gram-negative bacterium and was first isolated in 1967 by Bowman et al. (1). The cells are arranged singularly, consistently uniform, and readily grown to a high concentration in liquid culture. These characteristics make *B. diminuta* most commonly used for challenge testing of sterilizing-grade filter retention, as described in ASTM F838 and Parenteral Drug Association (PDA) Technical Report 26 (TR26) (2).

A combination of long- and short-sequence reads was used to obtain the complete genome sequence of the *B. diminuta* ATCC(B) 19146 strain. For DNA extraction, isolates were first grown on tryptic soy broth at 30°C for 48 h. For the Illumina sequencing, the genomic DNA was extracted using a plant genomic DNA kit (catalog number DP305; Tiangen), and concentration was quantified using a NanoDrop 2000 instrument (Thermo Scientific, Waltham, MA). Libraries were prepared using the TruePrep DNA library prep kit V2 for Illumina (Vazyme). Individual libraries were assessed on the QIAxcel advanced automatic nucleic acid analyzer and then were quantitated through quantitative PCR (qPCR) by the use of KAPA SYBR fast qPCR kits. Last, the library was sequenced on a HiSeq X Ten platform (Illumina, Inc., San Diego, CA, USA), and 150-bp paired-end reads were generated. For Nanopore sequencing, genomic DNA was extracted using a Gentra Puregene yeast/bact. kit (Qiagen, Valencia, CA). The DNA concentration was quantified and quality controlled using a Nanodrop 2000 instrument (Thermo Scientific, Rockford, IL) and Qubit fluorometer (Life Technologies, Grand Island, NY). Libraries were prepared using a 1D ligation sequencing kit (catalog number SQK-LSK108; Nanopore) and sequenced on the GridION system using a FLO-MIN-106 flow cell (Nanopore).

Oxford Nanopore GridION sequencing generated 66,507 reads (0.89 Gb) subsequently filtered with NanoFilt (minimum average read quality score, 7; minimum read length, 1,000) (3), with a mean Q score of 9.5 and an average length of 16,977 bp. Canu version 1.7 was used to correct the errors in the long-read sequencing with a minReadLength of 1,000, correctedErrorRate of 0.144, and other default parameters, resulting in an average depth of coverage of 180× for the chromosome (4). The HiSeq X Ten sequencer produced 3,793,859 paired reads, and combined assembly of the Oxford Nanopore and Illumina data was performed by using Unicycler version 0.4.5 under normal assembly mode with a 1,000-bp minimum fasta length; 65, 69, 73, 79, 85, and 95 kmers; and other default parameters, including a polishing step with Pilon (5). Genes were predicted using the NCBI Prokaryotic Genome Annotation Pipeline version 4.5 (6).

The genome of strain Brevundimonas diminuta ATCC(B) 19146 is in a circular chromosome of 3,551,819 bp, containing 3,375 protein-coding sequences (CDSs) and 61 RNAs, with a G+C content of 67.4%.

### Data availability.

The complete genome sequence of *B. diminuta* ATCC(B) 19146 was deposited in GenBank under the accession number CP035093. The raw sequences are available in the NCBI SRA database under the accession number PRJNA514930.

## References

[1] Bowman FW, Calhoun MP, White M 1967 Microbiological methods for quality control of membrane filters. J Pharm Sci 56:222. doi:10.1002/jps.2600560214. 5337945

[2] Parenteral Drug Association. 1998 Sterilizing filtration of liquids. Technical report no. 26. PDA J Pharm Sci Technol 52:1–31. 9693385

[3] De Coster W, D'Hert S, Schultz DT, Cruts M, Van Broeckhoven C 2018 NanoPack: visualizing and processing long-read sequencing data. Bioinformatics 34:2666–2669. doi:10.1093/bioinformatics/bty149. 29547981 PMC6061794

[4] Koren S, Walenz BP, Berlin K, Miller JR, Bergman NH, Phillippy AM 2017 Canu: scalable and accurate long-read assembly via adaptive k-mer weighting and repeat separation. Genome Res 27:722–736. doi:10.1101/gr.215087.116. 28298431 PMC5411767

[5] Wick RR, Judd LM, Gorrie CL, Holt KE 2017 Unicycler: resolving bacterial genome assemblies from short and long sequencing reads. PLoS Comput Biol 13:e1005595. doi:10.1371/journal.pcbi.1005595. 28594827 PMC5481147

[6] Tatusova T, DiCuccio M, Badretdin A, Chetvernin V, Nawrocki EP, Zaslavsky L, Lomsadze A, Pruitt KD, Borodovsky M, Ostell J 2016 NCBI Prokaryotic Genome Annotation Pipeline. Nucleic Acids Res 44:6614–6624. doi:10.1093/nar/gkw569. 27342282 PMC5001611

